# Polyethyleneimine Mediated DNA Transfection in Schistosome Parasites and Regulation of the WNT Signaling Pathway by a Dominant-Negative SmMef2

**DOI:** 10.1371/journal.pntd.0002332

**Published:** 2013-07-25

**Authors:** Shuang Liang, Matty Knight, Emmitt R. Jolly

**Affiliations:** 1 Department of Biology, Case Western Reserve University, Cleveland, Ohio, United States of America; 2 Biomedical Research Institute, Rockville, Maryland, United States of America; University of Pennsylvania, United States of America

## Abstract

Schistosomiasis is a serious global problem and the second most devastating parasitic disease following malaria. Parasitic worms of the genus *Schistosoma* are the causative agents of schistosomiasis and infect more than 240 million people worldwide. The paucity of molecular tools to manipulate schistosome gene expression has made an understanding of genetic pathways in these parasites difficult, increasing the challenge of identifying new potential drugs for treatment. Here, we describe the use of a formulation of polyethyleneimine (PEI) as an alternative to electroporation for the efficacious transfection of genetic material into schistosome parasites. We show efficient expression of genes from a heterologous CMV promoter and from the schistosome Sm23 promoter. Using the schistosome myocyte enhancer factor 2 (SmMef2), a transcriptional activator critical for myogenesis and other developmental pathways, we describe the development of a dominant-negative form of the schistosome Mef2. Using this mutant, we provide evidence that SmMef2 may regulate genes in the WNT pathway. We also show that SmMef2 regulates its own expression levels. These data demonstrate the use of PEI to facilitate effective transfection of nucleic acids into schistosomes, aiding in the study of schistosome gene expression and regulation, and development of genetic tools for the characterization of molecular pathways in these parasites.

## Author Summary

Schistosomiasis is a global disease infecting more than 240 million people worldwide and is ranked second only to malaria in global health importance. The causative agents of human schistosomiasis are parasitic worms that ingest red blood cells and can live for decades producing hundreds of eggs daily. There is one primary drug for treatment of schistosomiasis, but its use for over 30 years has raised concern over the development of drug resistance and thus created a need for new drugs. A challenge to the rational development of effective antischistosomals has been the difficulty in manipulating schistosome gene expression, and thus a limitation in our understanding of schistosome gene function. Here, we present a new and straightforward method for inserting genes into schistosomes and expressing them. In addition, to our knowledge we provide the first example of dominant negative gene expression to modify transcriptional regulation using a molecular genetics approach to study this globally important parasite.

## Introduction

The use of transgenesis and other technological advances has had a powerful impact in the molecular characterization and functional analysis of gene function in model organisms [1], [2]. However, like many parasitic worms, the natural characteristics of the schistosome (its complex life cycle involving multiple hosts, the absence of an immortalized cell line, and the inability to maintain the entire life cycle *in vitro*) have made in-depth genetic modifications challenging [3]. Schistosomes are the causative agents of human schistosomiasis, a parasitic disease that is endemic in 78 countries worldwide and that infects almost 240 million people [4]. In terms of morbidity and mortality, schistosomiasis is considered to be the most important helminth infection [5]. Although our knowledge of schistosome biology has increased over the last few years, the lack of simple and effective methodologies to manipulate schistosomes has slowed our understanding of schistosome molecular biology significantly behind other systems.

With the sequencing of the schistosome genome and recent updates to schistosome annotation [6], [7], research has focused on the functional analysis of schistosome genes. This includes approaches to insert DNA/RNA into schistosomes and to induce gene expression. Strategies used thus far for transfection of DNA/RNA molecules include the use of particle bombardment [8]–[10], soaking [11]–13, electroporation [14]–[18], chemical or lipofectamine based approaches [16], [19], and viruses [20], [21] (for review see [22]).

The insertion of genetic material into schistosomes by soaking in high concentrations of DNA/RNA has been successful for delivering siRNA and dsRNA. [11], [12], [19], [22], [23]. This approach is straightforward; however, the transfection efficiency is highly restricted by the size of nucleic acid fragment delivered, and worm death resulting from the use of highly concentrated nucleic acids [16]. In addition, this approach is not suitable for long-term modification of the parasite genome, which requires the use of compatible sized vectors that carry information for transcription, self-amplification, and the insertion of transposable elements.

The use of biolistic particle delivery for *Schistosoma mansoni* (*S. mansoni*) transfection has been successful for several developmental stages of the parasite. These include the adult, sporocyst, and miracidia [10], [24]–[26]. However, the square wave electroporation approach to introduce naked plasmid-based and non-plasmid-based exogenous genes into schistosomes has been more successful [8], [15]–[17], [27]. Square wave electroporation is more effective for transfection of schistosome eggs than the use of pseudotyped murine leukemia virus [21]. Consequently, electroporation has become the method of choice for schistosome transgenesis, specifically for the delivery of siRNA, dsRNA and vector based shRNA for gene silencing studies by RNAi [14]–[19], [23], [27]–[30]. One report, however, has suggested that the biolistic particle delivery method is more effective than electroporation for the delivery of RNA into adult worms and miracidia [8]. Nonetheless, both particle bombardment and electroporation can be damaging or even lethal to cells and parasites due to the physical damage or intense electrical charges, respectively [31], [32].

Polyethyleneimine (PEI) is a synthetic polymer with a highly cationic charge that can facilitate gene transfection in cells, and was identified as an oligonucleotide transfection reagent in 1995 [33]. PEI tightly binds to DNA by electrostatic interaction, induces DNA condensation and packaging into nanosized particles, and protects DNA from degradation, increasing efficiency for entrance into cell nuclei [34]–[37]. PEI as a transfection reagent is available in either linearized or branched structures and in a range of molecular weights. Several reports suggest that linearized PEI is the most efficient and optimized reagent for transfection, compared to the branched, higher molecular weight form [38], [39]. Here we report the use of PEI for the transfection of schistosomes.

To our knowledge, this is the first report of the use of PEI for schistosome transfection. The idea was inspired by the use of PEI to transfect the *S. mansoni* intermediate host, *Biomphalaria glabrata* (*B. glabrata*) [38]. Here, we evaluate the use of PEI as a transfection reagent for schistosomes using a plasmid encoding the mCherry fluorescent protein, and a Neomycin selectable marker. We then assess two RNA polymerase II (pol II) promoters for their ability to drive transcription of the reporter gene.

Previously, we characterized the schistosome myocyte enhancer factor (SmMef2) [40], a DNA-binding transcriptional activator that is important for cellular development, morphogenesis and survival in mammals and *Drosophila* (for review on Mef2, see [41]). We identified potential SmMef2 DNA binding elements in the promoters of wingless-type MMTV integration site family members 1 and 2 (*Wnt1* and *Wnt2*) homologs. WNT genes are conserved oncogenes that play a significant role in cell development, cell signaling and cell fate during early development [42]–[44]. Although the WNT pathway in schistosomes has not been extensively characterized, some WNT genes have been described in these worms [45]–[47]. We proposed that these two schistosome WNT genes homologs Smp_152900 (SmWnt1) and Smp_167140 (SmWnt2) could be potential targets of SmMef2.

Here, we developed a dominant negative form of SmMef2 (SmMef2,133) that lacks a transactivation domain and using this genetic mutant provide evidence that SmMef2 can regulate Sm*Wnt1* and Sm*Wnt2* gene transcription levels. Finally, we provide data supporting a role for SmMef2 in regulating its own transcription.

## Materials and Methods

### Animal preparation

Cercariae of *S. mansoni* NMRI strain (NR-21962) or strain PR-1 (NR-21961) were shed from the infected *B. glabrata* snails obtained from the Biomedical Research Institute (Rockville, MD) and transformed into schistosomula as previously described [48], [49] Seven to ten thousand schistosomula were cultured in complete RPMI medium (RPMI, 5% Fetal Bovine Serum, 1× Pen/Strep) per well in 12-well cell culture plates (Greiner Bio-One, Orlando, FL) at 37°C and 5% CO_2_ for 4 hours before being utilized for transfection. For the longevity experiment, modified Basch Medium 169 (Basch Medium 1695, Fetal Bovine Serum, 1× Pen/Strep) was used for the first three days of culture. After three days, the media was changed and replaced with complete Basch Medium [48].

### Construction of vectors for transfection

**Figure 1:**
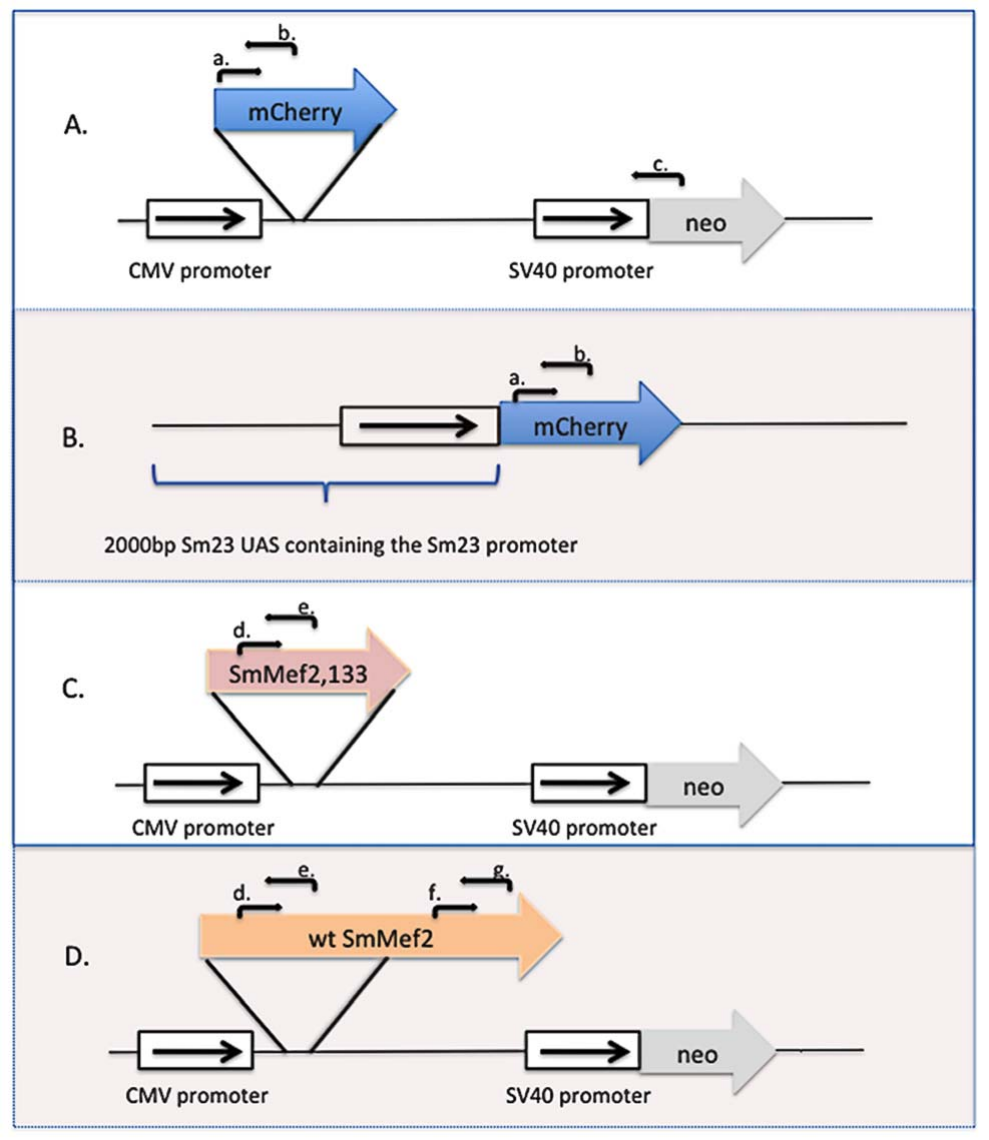
Expression constructs used for schistosome transfection. (A) The mCherry reporter gene was cloned into the BamHI site of the pCI-neo vector (Promega) to make plasmid pEJ1175. DNA oligos used for amplification by PCR or RT-PCR are shown as a forward arrow (a) or reverse arrows (b and c) representing forward oligo oEJ1022 (a), and reverse oligos oEJ1023 (b) and oEJ1019 (c). (B) 2000 base pairs of the Sm23 UAS was used to control expression of the mCherry and the Sm23 genes, These genes were cloned into the 7.4 kb pGBKT7 vector to make plasmid pEJ1116. (C) The N-terminal 133 amino acids of SmMef2 are regulated by the CMV promoter and were cloned to make plasmid (pEJ1181). The N-terminus of SmMef2 contains the DNA binding domain, but not its C-terminal transactivation domain. (D) The wild-type SmMef2, regulated by the CMV promoter, was cloned to make plasmid (pLS068). DNA oligos (d) and (e) are used for detection of SmMef2,133 transcript by qRT-PCR, while oligos (f) and (g) are used for specifically measuring wt SmMef2 transcript in qRT-PCR reactions.

DNA primers were designed and ordered from Integrated DNA Technologies (IDT, Coralville, IA). Subcloning was performed using the In-Fusion HD Cloning kit (Clontech, Mountainview, CA). The full transcript of the mCherry gene from the transposon vector pKM225 (GenBank: HQ386859.1), the first 399 bp (133 amino acids) of *SmMef2*, and the wild-type *SmMef2* [40] (NCBI accession number: JN900476) were amplified by PCR using Phusion High-Fidelity DNA Polymerase (NEB, Ipswich, MA) with three sets of primers: oEJ1020 forward (5′-TCA CGC GTG GTA CCT CTA GAA TGG TGA GCA AGG GCG AGG AG) and oEJ1021 reverse (5′-GCC CGG GTC GAC TCT AGA TTA CTT GTA CAG CTC GTC CAT GCC), oEJ1026 forward (5′-CAC TAT AGG CTA GCC TCG AGA TGG GTC GCA AAA AAA TAC TCA TC) and oEJ1027 reverse (5′-GCG TGA ATT CTC GAG CTA CGG TGT TTT AGT TCC TGT TCG TAT), and oLS197b forward (5′-ATA GGC TAG CCT CGA GAT GGG TCG CAA AAA AAT ACT CAT CA-3′) and oLS198 reverse (5′-TAA AGG GAA GCG GCC GCT CAA AGG TGG CGC ACA CGT TTA AGA-3′), respectively. mCherry and truncated Sm*Mef2* (Sm*Mef2,133*) amplicons were subcloned into the pCI-neo plasmid (Promega, Madison, WI) at the XbaI and XhoI sites, respectively. The wild-type Sm*Mef2* (Sm*Mef2*) amplicon was subcloned into the pCI-neo plasmid at the XhoI and NotI sites. Constructs were transformed into chemically competent One Shot TOP10 cells (Invitrogen, Carlsbad, CA). The mCherry reporter plasmid (pEJ1175), *SmMef2,133* expression plasmid (pEJ1181) and *SmMef2* expression plasmid (pLS068) (Figure 1) were purified using the Nucleospin Plasmid miniprep kit (Clontech, Mountainview, CA) and verified by restriction digestion analysis.

Plasmid pEJ1116 contains 2000 base pairs of the Sm23 upstream activation sequence (UAS) regulating the expression of mCherry. To make this construct, the mCherry transcript was amplified from plasmid pEJ604 using primers oJM16 forward (5′-CGT TTG AAA GTA TGG GAT CCA TGG TGA GCA AGG GCG AGG AG) and oJM17 reverse (5′-CTG TTT TCT TTG CAG TGT CTG CAG TTA CTT GTA CAG CTC GTC CAT GCC), then subcloned between the BamHI and PstI sites in the pGBKT7 vector (Clontech, Mountainview, CA). The 2000 base pair region containing the upstream activation sequence of Sm23 was amplified from schistosome genomic DNA using oligos oJM12 forward (5′-ATG GAG GCC GAA TTC CCG GGA CCC GAA CAC TAT AGT GTG ATG CAG) and oJM13 reverse (5′-CCG CTG CAG GTC GAG GAT CCC ATA CTT TCA AAC GGG ACA CAA TGC), then subcloned into the XmaI and BamHI sites of the same vector to make plasmid pEJ1116. To review, plasmid pEJ1116 contains the 2000 base pair UAS of the Sm23 promoter, followed by the mCherry reporter gene (Figure 1B).

### PEI-mediated plasmid transfection

InVitroPlex-Express-Parasite (Cat # IVTP-ExPA-002), a formulation of PEI optimized for nucleic acid delivery into parasites, was received as a gift from Dr. Puthupparampil Scaria (AparnaBio, Rockville, MD), and used for the transfection of schistosomes. PEI (7.2 µg) and DNA plasmid (4.8 µg), either pEJ1175, pEJ1181, or pEJ1116, were diluted in 1 mL of complete RPMI [49], separately. Then, the 1 mL PEI solution was added to the 1 mL DNA solution drop by drop to make a 2 mL PEI/DNA mixture with a PEI nitrogen and DNA phosphate (N/P) ratio of either 6∶1 or 11∶1, followed by 10–15 sec vigorous vortexing. The PEI/DNA RPMI solution was incubated at 37°C for 30 min to allow the PEI and DNA to form a nanoparticle complex. The complete RPMI from the 4 h schistosomula culture was carefully removed, leaving the schistosomula at the bottom of the culture well. Two mL of pre-warmed PEI/DNA solution was then added to the plate well and schistosomula were grown in the transfection mixture for another 40 h at 37°C and in 5% CO_2_. All above procedures were performed under sterile conditions. For each DNA transfection experiment, schistosomula were cultured in complete RPMI medium lacking PEI or DNA, or without both as negative controls.

### Total DNA/RNA extraction from schistosomula

At 40 hours post-transfection, the supernatant was removed from 44 h schistosomula by centrifuging the parasites at 1,500× g for 2 min. Recovered parasites were washed with 1.5 mL of 1× phosphate buffered saline (137 mM NaCl, 2.7 mM KCl, 4.3 mM Na_2_HPO_4_ and 1.47 mM KH_2_PO_4_ at a final pH of 7.4) twice to remove potentially contaminating residual DNA and PEI remaining in the tube, incubated for 15 minutes in 50 units of DNAse I to remove any remaining external DNA, and resuspended in 1× phosphate buffered saline.

Total DNA was purified using phenol-chloroform (Thermo Scientific, Waltham, MA). Five mg/mL glycogen (Invitrogen, Carlsbad, CA) and 3 M sodium acetate were added during the purification to increase the yields of DNA.

Total RNA was isolated following the standard manufacturer's protocol for the PureLink RNA Mini Kit using TriZol reagent (Invitrogen, Carlsbad, CA). DNase I digestion was performed to eliminate DNA contamination.

RNA and DNA were quantified on a Nanodrop 8000 spectrophotometer (Thermo Scientific, Waltham, MA) and the quality was verified by visualization on agarose gels.

### Transfection and gene transcript analysis

The transfection of vector pEJ1175 into parasites was tested by standard PCR using Taq DNA Polymerase (NEB, Ipswich, MA). 150 ng of total DNA was used as a template. Forward oligo oEJ1022 (5′-TAA CAT GGC CAT CAT CAA GGA GTT C) and reverse oligo oEJ1019 (5′-ATA CTT TCT CGG CAG GAG CA) were added to amplify a 2377 base pair DNA fragment including a partial mCherry and a partial neomycin sequence within the plasmid (Figure 1).

Total RNA from each DNAse treated sample was used to make cDNA by RNA reverse transcription reaction using SuperScript III Reverse Transcriptase, RNase OUT and oligo (dT)_12–18_ (Invitrogen, Carlsbad, CA) in a total volume of 20 µL volume. A no reverse transcriptase control was used in all experiments. The reaction was performed at 50°C for 40 min and then treated with 10 U RNase H (New England Biolabs, Ipswitch, MA) at 37°C for another 20 min to digest mRNA thoroughly. Both reverse transcriptase and RNase H were inactivated by incubation at 70°C for 15 min. The quality of cDNA was tested by PCR amplification of a 374 bp Sm23 gene fragment using primers oJM18 forward (5′-CGT TTG AAA GTA TGG GAT CCA TGG CAA CGT TGG GTA CTG GTA TGC) and oJM20 reverse (5′-GCC CTT GCT CAC CAC GGA TCC TTT GTA AAC AAC TGC AAC TAT GGC) (Supplementary Figure S1, Figure S2).

To analyze the expression of the mCherry gene under control of a human cytomegalovirus (CMV) promoter (pEJ1175, Figure 1A) and Sm23 promoter (pEJ1116, Figure 1B), qRT-PCR was carried out using primers oEJ1022 forward and oEJ1023 reverse (5′-TAC ATG AAC TGA GGG GAC AGG ATG T), to clone a 192 bp mCherry gene fragment from 60 ng cDNA template.

Two sets of primers were designed for detection of *SmMef2* transcripts by qRT-PCR. The first set of primers measure Sm*Mef2* within the first 399-base pair (the truncated region). The second sets of primers are located at the 3-prime end of SmMef2 (outside of the first 399 base pairs), and were used to detect wild-type Sm*Mef2* transcripts. The other primers sets were designed for detection of four Sm*Mef2*'s potential downstream targets. All primers were verified by Primer3 online software (http://frodo.wi.mit.edu/, Supplementary Table S1).

Sixty nanograms of cDNA from parasites treated with both PEI and pEJ1181 was used as template in a 60 µL qRT-PCR reaction with Power SYBR Green Master Mix (Applied Biosystems, Foster City, CA). Each reaction was divided into 20 µL triplicates and PCR was carried out and analyzed by StepOnePlus Real-Time PCR System (Applied Biosystems, Foster City, CA). This was done at least in triplicates for each sample. Transcript levels in schistosomes transfected with pEJ1175 and pLS068 were quantified by qRT-PCR, to differentiate between the non-specific gene amplification (mCherry) and wild-type Sm*Mef2* overexpression, as a result of overexpression SmMef2,133, respectively. Negative controls (No RT and DNA only treatment) were run in parallel. The qRT-PCR conditions were as follows: 95°C for 10 min, 45 cycles of 15 s at 95°C, 30 s at 60°C and 30 s at 72°C. The melt-curve analysis of each pair of primers showed that only one specific product was amplified by each reaction. The relative gene expression was calculated by the ΔΔC_t_ method according to the formula: expression rate = 2^−ΔΔCt^ and cyclophilin was used as an endogenous control gene [40]. Experimental data were verified by Student's t-test, and a p-value less than 0.05 was considered to be statistically significant [50]. Amplification efficiencies of target genes and the endogenous control gene, cyclophilin, are optimal and comparable.

### Protein expression and Western blot analysis

Both Sm*Mef2*,133 and the wild type Sm*Mef2* gene with a c-Myc tag at the 5-prime end were amplified from pEJ1114 [49] and subcloned into pCI-neo vector using the methods described above. Seven to ten thousand schistosomula transfected with one of the two c-Myc tagged plasmids and were harvested 44 h after cercarial transformation. Samples transfected with pEJ1175 were used as a negative control. Schistosomula were washed with 1× PBS twice and resuspended in the lysis buffer (20 mM Tris-HCl, 200 mM NaCl, 1× PMSF and 1× Halt Protease Inhibitor Cocktail; Thermo Scientific, Waltham, MA), followed by 6× sonication of 15 s pulses, 30% amplitude with 1 min interval between each pulse. Cell lysate was then added with 5× SDS loading buffer and boiled at 100°C for 10 min and incubation for 5 min on ice. Fifty microliters of the supernatant from each cell lysate was resolved on NuPAGE 4–12% Bis-Tris ready-made gels (Invitrogen, CA). The protein was transferred to a nitrocellulose membrane (Thermo Scientific, MA) and blocked in 5% milk. The specific expression of c-Myc tag protein was detected by the mouse monoclonal IgG_1_ c-Myc (Myc.A7) primary antibody and a goat anti-mouse IgG-HRP secondary antibody (Santa Cruz Biotechnology, CA). Similarly, mCherry protein expression was detected by the mouse monoclonal IgG2a primary antibody (Novus Biologicals, Littleton, CO) and the goat anti-mouse IgG-HRP secondary antibody (Santa Cruz Biotechnology, CA), and assayed by western blot analysis.

## Results

### PEI facilitates the transfection of DNA in schistosomes

**Figure 2:**
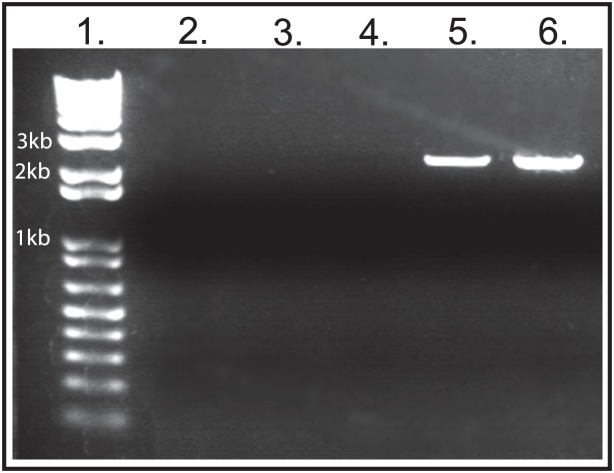
PEI is effective for the transfection of plasmid DNA into schistosomes. PEI was used to facilitate the uptake of plasmid DNA (pEJ1175, Figure 1A) into 4-hour schistosomula and analyzed for the presence of plasmid DNA after 40 hours by PCR amplification of a 2,377 bp plasmid DNA fragment. Each sample used 150 ng of total schistosome DNA extracted from schistosomes as a template to perform PCR, as seen here on a 0.8% agarose gel. Lanes 1–6 are as follows: 1] 1 Kb Plus DNA Ladder (Invitrogen, Carlsbad, CA); 2] Treatment with PEI only; 3] Treatment with plasmid DNA pEJ1175 only; 4] Untreated- no PEI, no plasmid DNA; 5] Treatment with both PEI and plasmid DNA, pEJ1175 with a N/P ratio of 6∶1; and 6] Treatment with both PEI and plasmid DNA, pEJ1175, with a N/P ratio of 11∶1.

PEI has been used successfully for gene delivery in mammalian cells *in vivo* and *in vitro* [51]–[53], and recently in the snail, *Biomphalaria glabrata* [38]. The success of gene delivery using PEI in snails inspired us to ask whether PEI could be used as an alternative to electroporation to transport genetic material into schistosomes. To test this possibility, we incubated 4-hour schistosomula for 40 hours in a PEI/plasmid DNA mix in complete RPMI (Figure 2, Lanes 5 and 6, see Material and Methods). The plasmid DNA contained the mCherry gene regulated by a strong CMV promoter (Figure 1A). As negative controls for transfection, equal numbers of schistosomula were cultured in RPMI medium containing (1) only PEI, (2) only DNA plasmid, or (3) only schistosomes, no PEI and no DNA (Figure 2, Lanes 2–4). We examined the efficacy of the use of PEI for the introduction of plasmid DNA into schistosomula by using different N/P ratios, 6∶1 (Lane 5) and 11∶1 (Lane 6). Forty hours after transfection, we treated all schistosomula with DNase to remove any contaminating external DNA. Total schistosome DNA was extracted from each group and used for standard PCR analysis to test for the presence of the 2,377 base pair fragment stretching from the mCherry gene to the neomycin gene of plasmid pEJ1175 (Figure 1A). We found that the expected 2,377 base pair fragment was amplified only from samples containing both PEI and plasmid DNA (Figure 2, Lanes 5 and 6), whereas all negative control samples (Lanes 2–4) had no product. This result is consistent and reproducible (n>5), and demonstrates that PEI can be used to introduce plasmid DNA into schistosomes.

After treatment with PEI, we observed the parasites by light microscopy. Under some conditions, PEI can have potential toxic effects to human cells [54]. To test for potential lethality to schistosomes due to PEI exposure, we incubated approximately 8,000 schistosomula in the 2 mL PEI∶DNA mix in complete RPMI media for two days. We found no significant differences in viability between schistosomula incubated with PEI (with plasmid DNA) and schistosomula grown without PEI in the medium (Supplementary Table S2). To assay viability, we pipeted the schistosomula in media and removed 5–10% of the parasite culture after 1 hour, 1 day, and 2 days. The schistosomula were allowed to settle briefly and were counted. We counted the schistosomula that settled on the culture dish and that were motile as alive, but schistosomula that did not settle on the plate, were not observed to be motile, or that appeared to lysed, were counted as dead. We rationalized that if PEI is deleterious, then under stressful conditions where the schistosomula were crowded due to large numbers, toxicity might be exacerbated. Our data indicate that lethality due to exposure to PEI is not a major concern for culturing schistosomula at the concentrations utilized in these experiments.

The nitrogen/phosphate (N/P) molar ratio of PEI∶DNA complexes is an important factor for effective transfection of DNA into mammalian cells [55], [56]. An N/P ratio of 6∶1 is optimal for the transfection of most mammalian cells [57]. Using PCR to amplify a 2,377 base pair sequence from plasmid pEJ1175 (Figure 1), we assessed whether a change in the ratio of PEI to DNA would affect the efficacy of the transfection of the plasmid DNA in schistosomes. We assayed two N/P ratios, 6∶1 PEI∶DNA, and 11∶1 PEI∶DNA, and both ratios were found to be effective for transfection of DNA into schistosomes (Figure 2, Lanes 5 and 6, respectively).

### CMV and Sm23 promoters can induce gene expression from a plasmid in PEI transfected schistosomula

**Figure 3:**
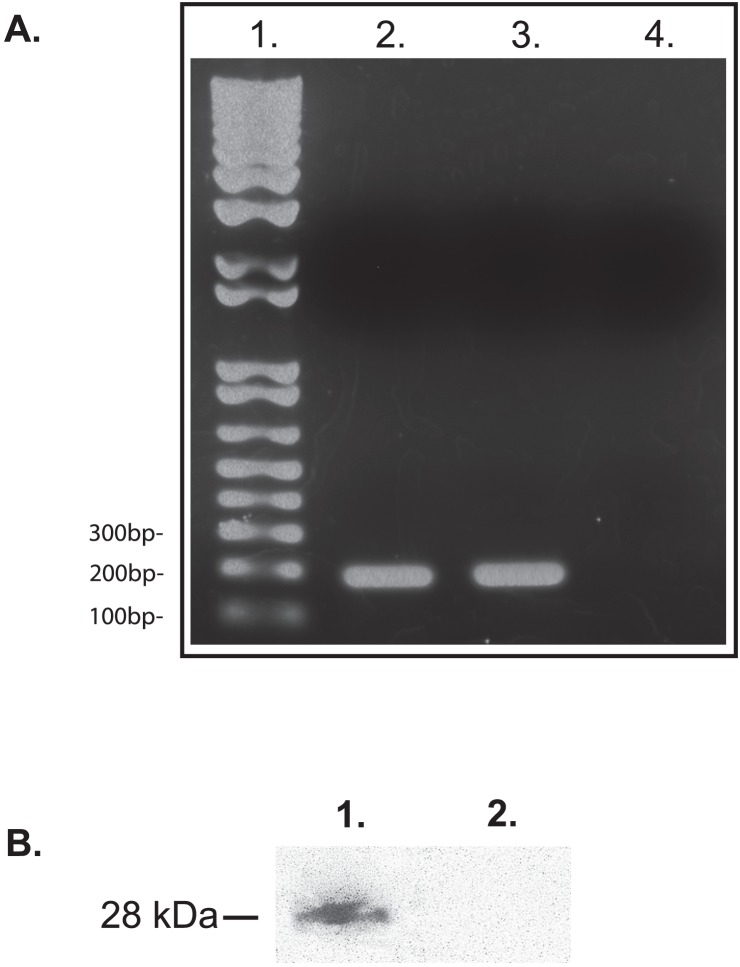
CMV and Sm23 promoters can induce gene expression from a plasmid in transfected schistosomes. mCherry gene expression is regulated by either the CMV promoter, or the schistosome Sm23 promoter. (A) RNA from each sample was extracted to produce cDNA using reverse transcriptase. Sixty ng of cDNA was used for PCR analysis to amplify a 192 bp mCherry gene fragment to test for mCherry transcript expression from either the CMV promoter or the Sm23 promoter. Lanes 1–4 from left to right are: 1] 1 kb Plus DNA Ladder (Invitrogen, Carlsbad, CA). 2] Sample treated with PEI and CMV promoter based vector pEJ1175. 3] Sample treated with PEI and Sm23 promoter based vector, pEJ1116. 4] Sample treated with PEI alone. (B) Total protein was extracted from schistosomes expressing mCherry under control of the CMV promoter (Lane 1) or from untransformed schistosome controls (Lane 2), and assayed by Western blot analysis using an antibody targeting the mCherry protein.

Since PEI can be used to insert DNA into schistosomes, we assayed whether the mCherry reporter gene, under control of a CMV promoter, could be expressed from a plasmid in transfected schistosomes. Previously, schistosomes transfected using either particle bombardment or electroporation showed that the CMV promoter is capable of inducing heterologous gene expression in these parasites [17], [58]. Thus, the use of CMV as a testable promoter was considered valid. To test for expression from the CMV promoter after PEI mediated transfection, DNase treated total RNA was extracted from schistosomula after treatment with or without PEI, and subsequently followed by two-step reverse transcription PCR (RT-PCR) to amplify a 192 base pair fragment of the mCherry RNA transcript (Figure 1A). Since mCherry is not endogenous in schistosomes, only parasites that have been successfully transfected with the plasmid will be capable of expressing mCherry. Our RT-PCR analysis confirms that the CMV promoter is sufficient to induce transcription of the mCherry reporter gene in schistosomula (Figure 3A, Lane 2), but not in the negative control sample (Figure 3A, Lane 4). No product was observed in a control sample tested without reverse transcriptase (data not shown).

We next evaluated whether a larger DNA plasmid could be transfected into schistosomes, and assayed the expression of a reporter gene on the plasmid directed by the schistosome Sm23 promoter. Sm23 is an integral membrane protein in schistosomes that is constitutively expressed during the schistosome life cycle [59], [60]. We cloned 2000 base pairs of the Sm23 upstream activation sequence containing the Sm23 promoter into the vector pGBKT7 (Clontech). Directly under control of the Sm23 promoter, we subcloned the mCherry gene (Figure 1B) to produce the 10.4 kb plasmid, pEJ1116 (Figure 1B). We showed that PEI could be used for transfection of the smaller 6.2 kb mCherry vector pEJ1175 (Figure 1A). Here, we evaluated PEI for the transfection of a larger 10,4 kb DNA plasmid. We transfected the 10.4 kb plasmid pEJ1116 into 4-hour schistosomula. After transfection of the 10.4 kb plasmid pEJ1116 into 4 hr schistosomula, we assayed for the amplification of a 192 base pair mCherry product to test for the expression of the mCherry transcript, as described above. Expression of the mCherry transcript can only occur in schistosomula that are successfully transfected and then, only if the plasmid based promoter, Sm23, is functional. After RT-PCR analysis, we found that mCherry is expressed from the Sm23 promoter on the 10.4 kb plasmid, demonstrating that PEI is sufficient to aid in the transfection of large plasmids into schistosomes, and that the Sm23 UAS is sufficient for gene expression from a plasmid (Figure 3A, Lane 3).

We investigated whether schistosomes transfected with a plasmid transcribing mCherry, under control of the CMV promoter, were able to express the mCherry protein using Western blot analysis (Figure 3B). Using an antibody against mCherry, we observed a 28 kD mCherry protein in schistosomes expressing mCherry from the CMV promoter (Figure 3B, Lane 1), but this was not observed in untransformed schistosomes (Figure 3B, Lane 2).

### Schistosome Mef2 is transcriptionally autoregulated and regulates WNT gene transcript levels

We previously identified and characterized Mef2 in schistosomes (SmMef2) [40], a conserved transcriptional activator that is essential for myogenesis in *Drosophila* [41]. Mef2 also has diverse functions regulating cellular differentiation, morphogenesis and proliferation [61], [62]. Recent studies in mice provide evidence that Mef2 proteins can modulate signaling of the WNT pathway during skeletal muscle regeneration [63]. We previously reported that there were potential Mef2 DNA binding sites within 500 bp of the translation start sites of two schistosome genes encoding WNT homologs: Smp_152900, encoding for *Wnt1*, and Smp_167140, which we assert, based on conserved sequence analysis, encodes *Wnt2* [64].

**Figure 4:**
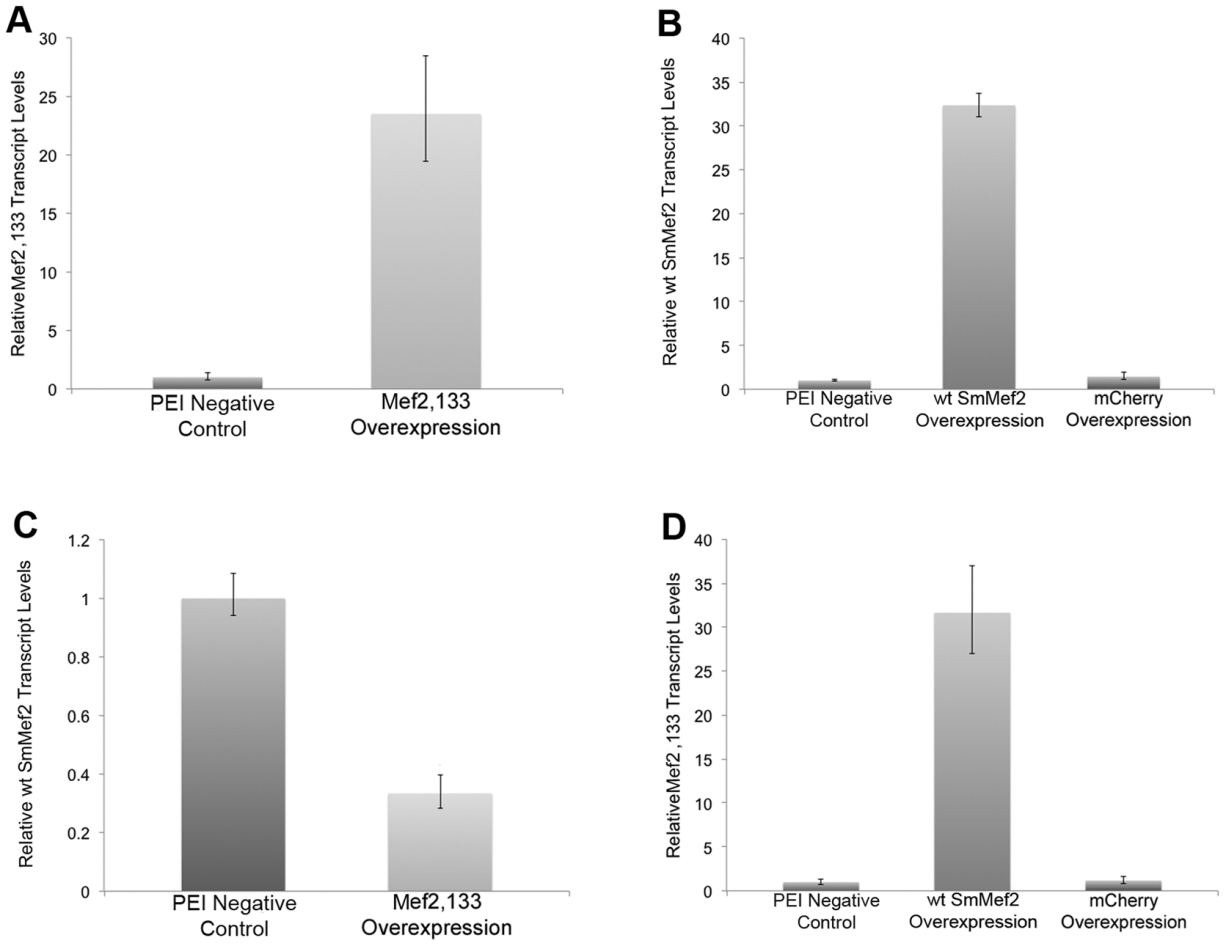
*SmMef2* can autoregulate its transcript levels. Schistosomula were transfected with a plasmid expressing SmMef2, the C-terminal deletion mutant containing the DNA binding domain of SmMef2 (Sm*Mef2,133*), or a negative control mCherry gene in the presence of PEI. Genes that are overexpressed (x-axis) are regulated by the strong CMV promoter. PEI Negative Control samples were exposed to the plasmid containing the SmMef2,133 mutant regulated by control the CMV promoter, but without PEI. qRT-PCR was used to analyze SmMef2 transcript levels or SmMef2,133 transcript levels (y axis) in response to overexpression of (A) the SmMef2,133, (B) SmMef2 (wt SmMef2 overexpression) or a mCherry negative control (mCherry overexpression), (C) SmMef2,133 transcript, and (D) and the wt SmMef2 transcript or a mCherry negative control.

The ability to easily transfect and induce gene expression in schistosomes with low lethality using PEI, and the developmental question of whether Mef2 plays a role in regulating genes in the WNT pathway, provided an opportunity to test whether schistosome transfection with PEI could be used as a genetic tool to dissect basic gene functions in schistosomes. To address this, we propounded the idea that expression of a SmMef2 mutant that can (1) bind DNA, but (2) be unable to efficiently induce Mef2 transcriptional target genes, could potentially interfere with normal SmMef2 activator function *in vivo* by acting as a competitive inhibitor and act as a potential genetic dominant negative in schistosomes. SmMef2 has a N-terminal DNA binding and a C-terminal transactivation domain. We removed the C-terminal transactivation domain of SmMef2, producing a truncation mutant comprising the first 133 amino acids containing the MADS box and Mef2 DNA binding domains to make SmMef2,133. We cloned the truncated schistosome *Mef2,133* gene so that its expression was controlled by the strong CMV promoter (Figure 1C) and transfected schistosomula with this construct as before. After 40 hours, we extracted RNA and used qRT-PCR to compare *SmMef2,133* transcript levels to an untransfected control. The control was incubated with the *Mef2,133* plasmid without PEI. We found that *SmMef2* levels were increased twenty-fold higher than *SmMef2* levels in the untransfected control (Figure 4 A), demonstrating significant upregulation of the *SmMef2* transcript.

**Figure 5:**
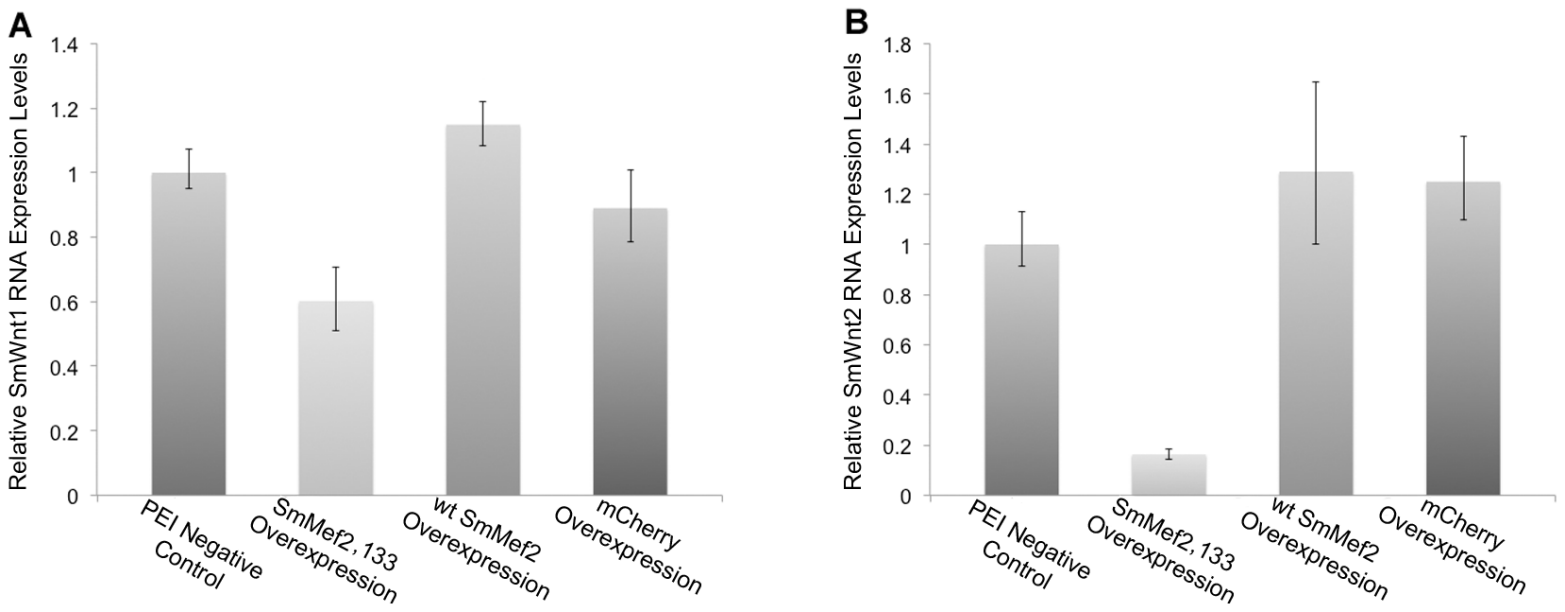
A dominant negative SmMef2 leads to downregulation of WNT genes in schistosomes. qRT-PCR analysis of (A) Wnt1 transcript levels and (B) Wnt2 transcript levels when transfected with plasmids expressing *SmMef2,133* (SmMef2,133 overexpression), wt SmMef2 (wt SmMef2 overexpression) and mCherry (mCherry overexpression). Genes that are overexpressed are regulated by the CMV promoter and compared to samples exposed to plasmid containing the SmMef,133 mutant in the absence of PEI (PEI Negative Control).

Since the promoters of *SmWnt1* and *SmWnt2* genes have Mef2 binding sequences, we tested whether SmMef2,133 overexpression has an effect on the transcript levels of *SmWnt1* and *SmWnt2* by qRT-PCR. When SmMef2,133 is overexpressed, we found that *Wnt 1* transcript levels are downregulated some 2 fold (Figure 5A), and *Wnt2* transcript levels were downregulated more than 5 fold compared to the untransfected control (Figure 5B). As a negative control, when mCherry was overexpressed we observed no significant changes in Wnt1 or Wnt2 transcript levels (Figure 5 A,B). When we tested a muscle LIM gene (Smp_143130) and a TGF beta family gene (Smp_063190) that have a potential Mef2 binding site, we found no significant difference in transcript levels (data not shown, Supplementary Table 1).

*SmMef2* levels are highest in 4-hour schistosomula relative sporocysts, cercariae, and adult worms [40]. We cloned the *SmMef2* gene under control of the CMV promoter, as was previously described for the truncation mutant, *SmMef2,133*, and overexpressed SmMef2.. To distinguish between expression of *SmMef2* and the mutant *SmMef2,133*, we designed DNA oligonucleotides that recognize *SmMef2* (Figure 1D, oligonucleotides f and g) but that do not recognize SmMef2,133 transcript. DNA oligonucleotides that recognize sequences in SmMef2,133 (Figure 1D, oligonucleotides d and e) also recognize SmMef2 sequences. We found that expression of *SmMef2* was elevated 30-fold relative to the control (Figure 4B), whereas overexpression of the mCherry negative control had no effect on *SmMef2* transcript levels. A second pair of oligonucleotides (Figure 1D, oligonucleotides d and e) that recognize sequences in *SmMef2,133*, and *SmMef2*, showed a 25 fold increase in Mef2 transcript levels (Figure 4D), indicating the confidence level of the qRT-PCR data.

We then assayed if overexpression of SmMef2 could have a positive effect on WNT levels. We found a very slight increase in *Wnt1* transcript, but no significant change in *Wnt2* transcript levels. The Mef2 protein is reported to positively regulate its own transcription [65]. We investigated whether SmMef2 was capable of regulating its transcription levels in schistosomes. To address this genetically, we overexpressed the truncated mutant, SmMef2,133, and measured *SmMef2* levels by qRT-PCR using oligonucleotides pairs that distinguish *SmMef2* transcript from *SmMef2,133* transcript as described (Figure 1D). We found that overexpression of SmMef2,133 resulted in a 3-fold decrease of *SmMef2* transcript (Figure 4C), strongly suggesting a role for Mef2 positively regulating its expression.

We assayed for distinct changes in viability between schistosomula expressing the dominant negative mutant, SmMef2. One thousand schistosomula were transfected with plasmid expressing *SmMef2,133*, SmMef2 or a nonspecific control, mCherry. These were grown for 7 days in Basch medium (see Material and Methods). After 7 days, all worms were quantified. We found 640, 550, and 620 schistosomula transformed with *SmMef2,133*, *SmMef2*, and the mCherry control respectively remained alive. Thus, we observed no significant differences in survival rate.

### SmMef2 protein is expressed in schistosomes

**Figure 6:**
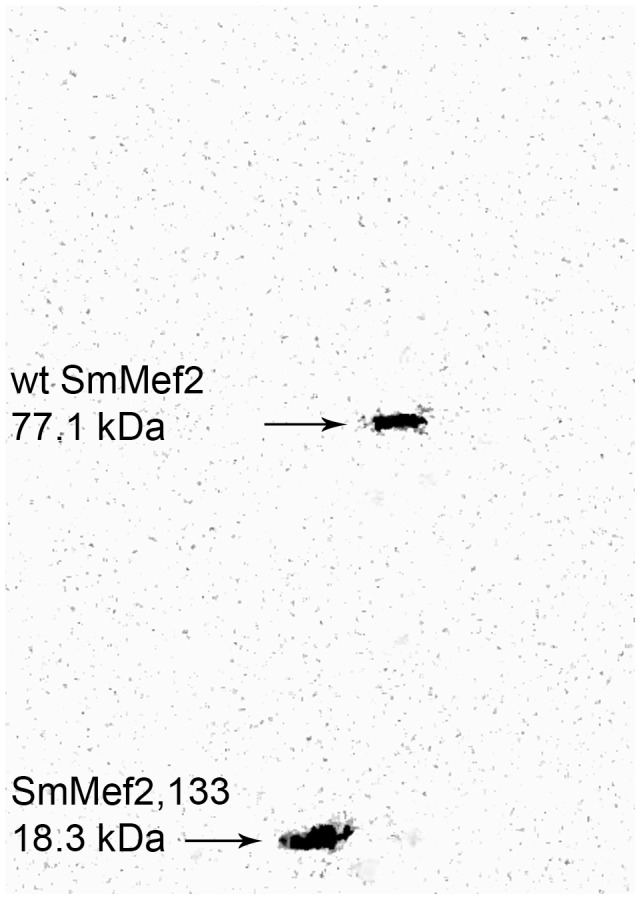
SmMef2 and SmMef2,133 transcripts are translated into protein in transfected schistosomes. SmMef2 and SmMef2,133 were cloned and N-terminally Myc-tagged to assay whether expression from the plasmid produced protein products. Proteins was assayed by Western blot Analysis of protein extracts from schistosomula 40 hours after transfection using anti-c-Myc monoclonal antibodies. Samples were visualized after exposure on a gel documentation system. The lower band (Lane 1) shows expression of the 18.3 kDa c-Myc tagged SmMef2,133 protein from the schistosomes transfected with the corresponding gene. The upper band (Lane 2) shows the 77.1 kDa c-Myc tagged wild-type SmMef2 protein expressed by the transfected schistosomes.

*SmMef2* and *SmMef2,133* transcript levels are upregulated when expressed from the CMV promoter. We addressed whether this expression led to production of protein. To assay protein expression from the reporter construct in schistosomes, we added a c-Myc tag sequence to the 5-prime ends of the truncated mutant *SmMef2,133* and the wt *SmMef2* genes. We then extracted protein from schistosomula transfected with the plasmids expressing myc tagged SmMef2 and myc-tagged SmMef2,133 at 40 h post-transfection, and we assayed protein expression by Western blot analysis. Both c-Myc tagged SmMef2,133 (18.3 kDa) and c-Myc tagged SmMef2 (77.1 kDa) were detected by Western analysis and visualized using a gel documentation system (Figure 6). These data confirm that exogenous gene transcripts are translated into protein.

## Discussion

Here, we have shown 1) that PEI facilitates the transfection of nucleic acids into schistosomes, and 2) that it facilitates the molecular genetic analysis of signaling and transcriptional pathways in schistosomes, addressed here by assessing SmMef2 function on *SmWnt1* and *SmWnt2* genes as proof of principle. 3) We provide an example of dominant-negative gene expression in schistosomes, and 4) provide evidence that SmMef2 is autoregulatory, and show data supporting its role in the regulation of the WNT pathway.

The idea to examine PEI for the transfection of DNA into schistosomes was inspired by the report that showed it was a useful agent for the successful transfection of *B. glabrata* snails [38]. PEI is an established transfection agent for individual mammalian cells [33], for tissue culture [66], and for tumor therapy [67]. The transfection of live snails led us to test whether it could also be efficacious as a transfection agent in schistosomes. We found that PEI (Aparna Biosciences, Rockville, MD) is extremely effective for the transfer of nucleic acids into schistosomula. DNA plasmids up to 10.4 kb in size were successfully introduced into schistosomula and were functional for transcription of a heterologous reporter. We recommend the use of PEI as an alternative to the aforementioned transfection approaches previously used in schistosomula. Although electroporation has been the most widely used method for transfection in schistosomes, electroporation can lead to significant mortality after passage of electrical amperage into worms. It also requires the purchase of an electroporator and cuvettes. The schistosomes must be transferred into cuvettes with nucleic acids in minimal salt solution to avoid arcing, prior to *in vitro* or *in vivo* culturing, increasing the possibility of contamination. Our data suggests that PEI at the levels used and described here does not increase lethality to transfected parasites. In addition, the use of PEI as a transfection agent is straightforward, requiring the addition of PEI and less than 10 µg of DNA in our studies. Mechanistically, DNA and PEI are incubated in the same culture medium containing the parasites, making the technique simple.

We tested two different promoters for expression in schistosomes after transfection using PEI- the CMV promoter, and the schistosome Sm23 promoter. Both promoters were capable of inducing gene expression from plasmids when tested 2 days after transfection was initiated. Initially, we used mCherry as a reporter gene for expression under the premise that we could visually screen for transfected schistosomula under a microscope and that we could be able to determine the exact efficiency of transfection by quantifying the percentage of fluorescent schistosomes. We found that the background autofluorescence of schistosomes masked consistent discrimination between transfected and untransfected schistosomes. Since the PEI does not specifically localize DNA during transfection to a discrete locations in the parasite (i.e. the gut, the nerves, schistosome surface) and there is as yet no organelle specific reporter described in schistosomes, it is possible that diffuse fluorescence of the mCherry reporter cannot be observed visually using our methods. Thus, we assayed transfection and reporter activity by directly quantifying schistosome RNA levels in the transfected parasites.

The ease of this approach to transfect schistosomes in combination with our interest in transcriptional regulation and our previous work on SmMef2 in schistosomes, stimulated us to inquire if we could develop a genetic model to investigate basic biological questions on *SmMef2* gene expression in schistosomes. We predicted that expression of a truncated SmMef2 protein, that contains the DNA binding domain but no transactivation domain (SmMef2,133), could antagonize or compete with wild-type SmMef2 for binding to SmMef2 transcriptional targets, and potentially interfere with expression of SmMef2 target genes. We identified potential Mef2 binding elements in several schistosome promoters, including *SmWnt1* and *SmWnt2* [40] When we overexpressed the truncated SmMef2,133, we found that both *SmWnt1* and *SmWnt2* transcript levels were reduced by two-fold and five-fold, respectively. We similarly overexpressed a control mCherry gene to test whether overexpression of any gene could lead to general down regulation of schistosome gene expression, but observed no change in Wnt1 or Wnt2 transcript levels. This indicates that Wnt1 and Wnt2 transcription is regulated by SmMef2, either directly or indirectly. Although, the presence of Mef2 binding sites in the promoters of *SmWnt1* and *SmWnt2* might suggest that this interaction is direct.

We overexpressed SmMef2 to assay whether elevated levels of SmMef2 levels could lead to an increase in *Wnt1* or *Wnt2* transcript levels. We found an indication of change in *Wnt1* transcript levels. *Mef2* transcript levels are highest in schistosomula compared to sporocysts, cercariae, or adults. It could be that the normal high expression level of *SmMef2* at this stage saturates Mef2 targets and increasing Mef2 levels higher has little effect. This rationale corresponds to work done on myoblast cells where a dominant negative version of Mef2 reduces MyoD induced myogenic colony formation, but overexpression of Mef2 had no effect on myogenic conversion [68]. Alternatively, it could simply be that Mef2 requires an interacting factor for the expression of some Mef2 targets or posttranslational modification of SmMef2, which has been established for several Mef2 target genes [69]–[73].

**Figure 7:**
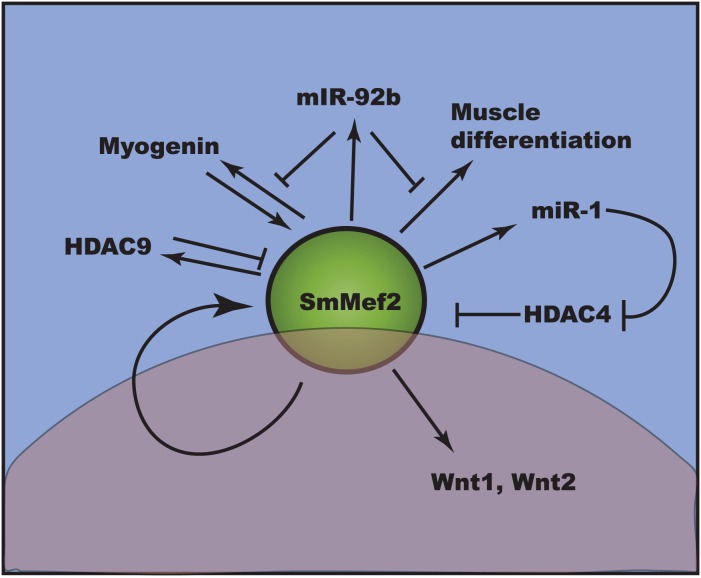
A simplified expanded model Mef2 gene regulation. Regulation of Mef2 is complex. Mef2 expression is induced by myogenin, and Mef2 protein induces myogenein and its own expression. Mef2 also regulates genes necessary for muscle differentiation and the microRNA miR-1. miR-1, in turn, inhibits the histone deacetylase HDAC4 from repressing Mef2, allowing increased Mef2 expression. Mef2 also induces HDAC9 and miR-92b, which work to negatively repress Mef2 expression and Mef2 activator function, respectively. The upper panel in blue describes known Mef2 interactions in other systems. The lower panel in pink highlights a role for SmMef2 in the regulation of SmWnt1 and SmWnt2, and in SmMef2 autoregulation in schistosomes as described in this report.

We also found that SmMef2 was capable of regulating its own transcript levels in whole schistosomes, which has been reported previously in mammalian cell culture [65]. When the dominant negative SmMef2,133 was overexpressed, *SmMef2* transcript levels were reduced three fold, showing that SmMef2 regulates its own transcription. In *Drosophila*, Mef2 interacts with microRNAs (miRNA), specifically miR-1, that targets and reduces the mRNA stability and translation of class II histone deacetylases (HDACs), specifically HDAC4. HDAC4 is a transcriptional repressor of muscle specific genes [74]. In this model, downregulation of Mef2 would reduce expression of miR-1. With reduced miR-1, HDAC levels are not suppressed and in turn can repress of SmMef2 (Figure 7). Thus, overexpression of Mef2 could presumably lead to an exponential increase in its expression. On the contrary, Mef2 also induces targets that negatively regulate its levels. Mef2 activates the miR-92b, a recently identified microRNA that represses *Mef2* transcript [75]. Similarly, Mef2 can activate HDAC9. HDAC9 in turn interacts with Mef2 proteins to repress Mef2 transcriptional ability [76]. Thus, Mef2 forms a feedback loop that maintains an equilibrium in Mef2 expression and Mef2 target induction. This may also explain why overexpression of SmMef2 did not produce a significant increase in Wnt1 and Wnt 2 expression levels.

Since Mef2 is important for myogenesis and neuron survival in other organisms, we predicted that overexpression of Sm*Mef2,133* would produce a phenotypically distinct mutant due to lack of muscle development or a reduction in neuronal survival. However, after microscopic analysis, we unable to identify a visual physical difference in either motility or in shape between schistosomes overexpressing *SmMef2,133*, or SmMef2, or a mCherry control schistosomula, even after 7 days. Nor did we find a quantitative change in a predicted muscle Lim gene (Smp_143130) or a TGF beta family gene (Smp_152900) at two days, which have a potential SmMef2 binding site (data not shown). One reason for this could be that factors other than SmMef2 can participate in muscle or neuromuscular development [41], [77]. Alternatively, the transcription activation function of SmMef2 protein function may be inhibited by a HDAC9-like protein in schistosome preventing induction, or simply that the schistosomula should be cultured for a longer periods to observe any gross phenotypic changes contributed to overexpression of *SmMef2,133*. Nonetheless, these data, and recently published data in mice, corroborate that Mef2 plays a role in regulating the WNT pathway, a connection that has not been extensively explored.

In *Drosophila* and in mammals, Mef2 activates genes that participate in the Notch-Delta, Hedgehog, Fibroblast Growth Factor and Epidermal Growth Factor pathways [41]. This report adds the WNT pathway to that list (Figure 7). It will be of interest to further examine the role of SmMef2 in these pathways in schistosomes.

The use of PEI for transfection is a simple tool that can be used to dissect schistosome genetic pathways. We have not yet tested whether it facilitate the transfection of nucleic acids into other stages of schistosome development, nor have we made a direct comparison between PEI transfection and electroporation, which could be informative. The successful use of PEI for the transfection of mammalian cells in culture, for whole snails, and for schistosomula, provides promise that this approach may work in other schistosome stages, and that it could be successfully used for other flat or roundworm species that have been challenging to transfect. In addition, we are currently evaluating commercial and noncommercial promoters for their ability to drive gene expression in schistosomes, using PEI for nucleic acid delivery. Eventually, we expect that schistosome expression vectors could be selected based on promoter transcription rates, stage-specific or location dependent expression, or for use as cellular markers. In addition, since PEI is thought to function by protecting nucleic acid from digestion [35], RNA interference constructs may be potentially used for transcript knockdown by using this approach.

## Supporting Information

Figure S1 **cDNA quality test by amplification of Sm23 gene fragment.** A 374 bp Sm23 gene fragment was PCR amplified from 20 ng of cDNA of each sample. Lane 1–4 from left to right are: 1. 1 Kb Plus DNA Ladder. 2. Sample treated with PEI and pEJ1181. 3. Sample treated with PEI and a mixture of pEJ1116. 4. Sample treated with PEI and pEJ1175. 5. Sample treated with PEI alone. 6. Sample treated with pEJ1181 alone. 7. Blank control treated without both PEI and vector DNA, respectively.
(EPS)

Figure S2 **No-RT control visualized on agarose gel after quantitative-PCR amplification.** A 246 bp SmMef2,133 gene fragment was amplified by quantitative RT-PCR from either cDNA templates or RNA templates of each sample. Lane 1–7 from left to right are: 1] 1 Kb Plus DNA Ladder; cDNA templates used for quantitative-PCR from samples transfected with 2] mCherry (pEJ1181), 3] wt SmMef2 (pLS068) and 4} SmMef2,133 (pEJ1175), respectively; 5–7] RNA templates used for quantitative-PCR from samples transfected with 5] pEJ1181, 6] pLS068, 7] pEJ1175
(EPS)

Table S1 **Gene names and primer sequences used for quantitative PCR analysis.** Gene names and DNA oligonucleotide sequences used for qRT-PCR analysis
(DOC)

Table S2 **PEI does not deleteriously affect schistosome survival under conditions used for transfection.** Survival rate of schistosomes was assayed over a two-day period in the presence or absence of PEI in RPMI complete media. Viable schistosome number was quantified at 1 hour, 1 day, and 2 days.
(DOC)

Table S3 **The potential downstream targets of SmMef2 picked for expression test.** Potential targets of SmMef2 tested for transcript level variations after overexpression of SmMef2,133
(DOC)
