# Supplementary material for: Polyethyleneimine Mediated DNA Transfection in Schistosome Parasites and Regulation of the WNT Signaling Pathway by a Dominant-Negative SmMef2
Source: PLoS Negl Trop Dis. 2013 Jul 25;7(7):e2332. doi: 10.1371/journal.pntd.0002332 (PMC3723562; doi:10.1371/journal.pntd.0002332)
Supplement: Table S1 — Gene names and primer sequences used for quantitative PCR analysis. Gene names and DNA oligonucleotide sequences used for qRT-PCR analysis (DOC) [file pntd.0002332.s003.doc]

**Supplemental Table S1.** **Gene names and primer sequences used for quantitative PCR analysis**

| **Primers for qRT-PCR** | | | |
| --- | --- | --- | --- |
| **Gene name (type)** | **Smp number** | **Forward primer** | **Reverse primer** |
| Cyclophilin | Smp_054330 | TGGGCGGATTTCATAAAGAC | TAAGCATCCAGTGCCAATGA |
| Truncated SmMef2 | Smp_129430 | GACGAACGAAATCGTCAGGT | TTTGCCTTCTTTACGGTTGA |
| Wild-type Sm*Mef2* | Smp_129430 | ATCTGTTTCAATGGCACTGG | ATGAACTTGGCGCGTATGTA |
| TGF beta family | Smp_063190 | TCGTCCACGTTTAAAAAGACA | CCTCGACAATAATTCGGTTCA |
| Muscle LIM | Smp_87250 | GGAGGTTCTGGAGCATTGAA | CAAGTGATTTCCCGCAAACT |
| SmWnt1 | Smp_152900 | AAACCCATCTCAACCACAGC | GCCGGAGTTGTCTGAATTGT |
| SmWnt2 | Smp_167140 | ATATGGGCAATGCTTGAAGG | ACAACACCAGCTGACCATGA |
