# Supplementary material for: Polyethyleneimine Mediated DNA Transfection in Schistosome Parasites and Regulation of the WNT Signaling Pathway by a Dominant-Negative SmMef2
Source: PLoS Negl Trop Dis. 2013 Jul 25;7(7):e2332. doi: 10.1371/journal.pntd.0002332 (PMC3723562; doi:10.1371/journal.pntd.0002332)
Supplement: Table S2 — PEI does not deleteriously affect schistosome survival under conditions used for transfection. Survival rate of schistosomes was assayed over a two-day period in the presence or absence of PEI in RPMI complete media. Viable schistosome number was quantified at 1 hour, 1 day, and 2 days. (DOC) [file pntd.0002332.s004.doc]

**Supplemental Table S2. PEI does not deleteriously affect schistosome survival under conditions used for transfection**

| Transfected plasmid | Survival rate (%) | | |
| --- | --- | --- | --- |
|  | 1-hour | 1-day | 2-day |
| No PEI+ mCherry (4.8 ug) | 77.3 | 69.8 | 35.0 |
| PEI (7.2 uL)+ mCherry (4.8 ug) | 81.2 | 63.5 | 32.8 |
| PEI (7.2 uL)+ SmMef2,133 (4.8 ug) | 70.5 | 58.9 | 34.6 |
