# Supplementary material for: Polyethyleneimine Mediated DNA Transfection in Schistosome Parasites and Regulation of the WNT Signaling Pathway by a Dominant-Negative SmMef2
Source: PLoS Negl Trop Dis. 2013 Jul 25;7(7):e2332. doi: 10.1371/journal.pntd.0002332 (PMC3723562; doi:10.1371/journal.pntd.0002332)
Supplement: Table S3 — The potential downstream targets of SmMef2 picked for expression test. Potential targets of SmMef2 tested for transcript level variations after overexpression of SmMef2,133 (DOC) [file pntd.0002332.s005.doc]

**Supplemental Table S3.** **The potential downstream targets of SmMef2 picked for expression test**

| **Putative gene type (name)** | **Smp number** | **Mef2 Binding site sequence** | **Distance upstream** |
| --- | --- | --- | --- |
| Muscle LIM | Smp_143130 | CTTTATTAG | 169 |
| TGF beta family | Smp_063190 | CTTAAAATAG | 310 |
| SmWnt2 | Smp_167140 | CTATAAATAA | 456 |
| SmWnt1 | Smp_152900 | CTATAAATAG | 433 |
